# “Drugs are a taboo”: a qualitative and retrospective study on the role of education and harm reduction strategies associated with the use of psychoactive substances under the age of 18

**DOI:** 10.1186/s12954-021-00481-9

**Published:** 2021-03-17

**Authors:** Gabriela Almeida Pinto da Silva, Catarina Pinto Pereira, Marta Sofia de Sousa Pinto

**Affiliations:** Faculty of Psychology and Educational Sciences, University of Porto, Porto, Portugal

**Keywords:** Psychoactive substances, Qualitative, Young people, Drug education, Intervention, Harm reduction, Retrospective study

## Abstract

**Background:**

The use of psychoactive substances frequently starts at a younger age than adulthood. Considering the perspective of young people, this retrospective study tried to provide them with a role in identifying their own needs regarding drug use and interventions focused on it; the obstacles in accessing both general health and harm reduction services; the changes needed for a more effective, congruent and empowering response to the use of psychoactive substances before the age of 18.

**Methods:**

The study was divided into two parts having both parts a qualitative focus. In the first part of the study, an online questionnaire was sent to all harm reduction teams and two focus groups were carried out with eight harm reduction professionals and six people who use(d) drugs. The second part used an online questionnaire applied to 143 participants aged between the age of 18 and 25 complemented by two semi-structured interviews.

**Results:**

The need for education about psychoactive substances, namely in the educational context; the lack of information about available services; and the need for confidentiality were the most mentioned issues by the young people. Also, the informal network played a significant role in the participants relationship with psychoactive substances.

**Conclusions:**

The lack of information was the most highlighted obstacle. Also, there is a confluence of various vulnerabilities such as being under 18, using drugs and the different social situations that they live in. To improve policy and practice regarding drug use among young people, harm reduction interventions must be scaled-up as well as intentionally driven to empower youth to deal with. School environment was chosen by participants as one of the elected environments to implement harm reduction services.

## Background

The use of psychoactive substances, classified generally into licit or illicit, has a long path in human history [1]. However, this distinction and the degree of repression exerted on each substance are not the result of a scientific assessment of the potential damages and benefits but of political ideologies [1]. Considering this, the term “drug” or “psychoactive substances” used in this article refers to both.

Adolescence is a privileged time for experimentation and risk behaviours such as using psychoactive substances [2–5] as reflected in many of the statistical studies [6, 7]. The adolescent is biased by more mature limbic regions to less mature prefrontal regions [8], continuously referred as an important region for cognitive control [8, 9]. The strengthening of connections within these two regions may provide an explanation for the changes in impulsivity and risk taking observed across development [9].

Using drugs is considered a risk behaviour as it can compromise, directly or indirectly, the psychosocial aspects of the adolescents’ development [3, 10, 11]. However, this behaviour cannot be seen as problematic per se due to the fact that not all drug use in adolescence will become problematic or chronic [10, 12–14]. In that sense, there is the need to explore holistic approaches that avoid censorship and pathologization [11, 12, 14] while accommodating two functions of drug use that should not be ignored: exploration and experimentation [2, 12, 15]. Hence, drug use may play a developmental function that must be taken into consideration in interventions with young people [11–16]. Seeing it as an exploratory behaviour gives the opportunity for the adolescent to have a participative role in their social environment [17] encompassing organizations that work with this target group [18, 19]. The adolescents' views about institutions and services reveal some obstacles towards their intervention. The most mentioned obstacle is confidentiality [20, 21] that when breached can lead to a feeling of mistrust in the institution [22–24]. Also, there is some evidence of a lack of information on how and where community services can be reached [16, 24]. These conclusions may unfold the lack of health literacy, an important tool to empower people to use and access effectively the information in order to have more control over their health [25, 26].

Being adolescence a privileged period for developing interventions that promote an empowered and an informed decision-making [27, 28], a possible solution is to invest in health education during this development stage [25, 26, 28–30]. Schools, as an organization that reaches a larger number of adolescents, may be one of the most adequate contexts to promote health literacy [28] in various domains such as drug use. One of the promising strategies to address it is thought to be harm reduction philosophy [15, 31–33] since it allows a more pragmatic and flexible response to drug use in adolescents [15, 31–33], and it can become a link between them and other health and social services [34–36].

Despite these advantages, young people's attendance to harm reduction services is reduced [16, 35, 37] due to the lack of knowledge about its existence [16]; the lack of adaptation to adolescents' drug use (e.g. smoking drugs vs injecting) [16, 34, 36, 37]; and the resistance of young people to assume themselves as people who use psychoactive substances [16, 36]. Also, age restrictions [16, 21, 34, 35, 37] prevent the professionals from retrieving important information and it imposes an ambiguous role to their intervention with this public [21, 34].

Hence, adolescents are seen as a vulnerable population due to their age [38], but in reality, their vulnerability is systemic [34, 37, 38] because they are excluded from the provision of services that can be a gateway for intervention [38]. In addition, they are not heard in issues that concern them [22, 39] such as in drug use [40, 41].

In order to encompass the complexity of the problems that adolescents face and to design interventions that are relevant and congruent with their sociocultural characteristics [20, 27, 31] it is necessary to give the key actors of this phenomena a primary role in the research. This was the goal in this paper where the adolescents took up the expert role in identifying their own needs, the obstacles in accessing and using general health services, harm reduction services and in the development of possible changes in the field of drug use in adolescence to build a more effective, congruent and empowering response.

For the purpose of this study, there was a division of the research questions^1^ in two parts. In the first part, we sought to explore this phenomenon in Portugal giving voice to the experienced harm reduction professionals and people who use(d) drugs above the age of 18 based on the following research question:

1. From their perspective, how do they portray the phenomenon of the use of drugs by young people under the age of 18 having in consideration the dimension of this issue, the needs and the obstacles the adolescents face when using harm reduction services?

In the second part of this study, we sought to explore this phenomenon in Portugal through the lens of the young people based on the following research questions:

2. From the adolescents´ perspective:
  - How is education about drugs presented in the formal and informal contexts in Portugal?
  - How could their needs when using drugs under the age of 18 be answered?
  - What is their vision about the services and institutions that they had some contact with?
3. From the adolescents' perspective, what is the relevance of the harm reduction philosophy for the young people who use drugs and how can this be adapted to be an interventional response for this target group?

## Method

### Methodology

When exploring this phenomenon, a gap was found in the literature: only few studies give emphasis to the perspective of the young people about issues that concern them and their insights for the alteration, improvement and development of interventions. Hence, this study undertakes a qualitative focus that promotes a thick and rich description of the individual perspectives, experiences and meanings, highlighting the existence of multiple realities that emerge according to the various contexts of experience of the individuals [42–44]. Also, this methodology allows the research to give voice to people in more vulnerable positions such as adolescents who used drugs under the age of 18 [44].

### Data collection—instruments and methods

The study was conducted at two different moments in time. In the first part of the study and with the intention of responding to the first research question, an online questionnaire and two focus groups were the selected instruments. The online questionnaire was chosen in order to retrieve some information about the use of drugs in adolescents through the perspective and the work experience of the harm reduction teams.^2^ On the other hand, the focal group was chosen due to its capacity to assess various perspectives about the same phenomenon through the interaction and discussion of the various participants [43].

The second part of the study used semi-structured interviews and another online questionnaire. These two instruments had the goal to retrieve information for the other two research questions that focus on the perspective of the adolescents. The online questionnaire was chosen due to the pandemic situation that prevented the researchers to be physically present to conduct the interviews on this sensible topic. Moreover, this instrument served as a connection between the participants and the researcher to hold the interviews by telephone. Although there were only two interviews collected, the data provided allow us to contextualize and triangulate the material from the other sources.

### Participants: recruitment and characteristics

This study comprised of different sample recruitment. In relation to the online questionnaire for the harm reduction teams, this was sent to all of them through SICAD (General Directorate for Intervention on Addictive Behaviours and Dependencies), a national organisation responsible for the coordination of all harm reduction teams. From 36 projects, 26 of those responded. Regarding the focus groups, the sampling was purposive in order to select the participants on the basis they will provide meaningful information [43, 44]. There was a focus group in the north of the country and another in the centre. The one conducted in the north was composed of two technicians with decision-making competence in the context of addictive behaviours, two people who use(d) drugs older than 18 and three harm reduction professionals. The focus group in the centre was constituted of one technician with decision-making competence in the context of addictive behaviours, four people who use(d) drugs older than 18 and two harm reduction professionals.

Lastly, the sample for the other online questionnaire was, initially, a convenience sample because adolescents who use drugs are hard to reach [43, 44]. Afterwards, it was used the snowballing technique where the sample was built through the initial participants enabling a total of 143 participants. The mean age was 20.88 and 67.8% of the participants were female. Also, 46.2% of the participants used drugs before the age of 18 with a higher prevalence being male (60%). The two interviews derived from the online questionnaire. One of the interviewees was a male aged 18, and the other one was a female aged 21. The inclusion criteria for the online questionnaire was aged 18–25 years old and for the interviews was aged 18–25 years and the use of drugs before the age of 18. The ethical and legal specificity of our country does not allow the participation of young people below the age of 18 without the parental authorization, and being drug use a sensitive and hidden behaviour, it was decided to do a retrospective study.

### Analyses

In relation to the quantitative data obtained in the online questionnaires, it was only conducted a descriptive analysis using SPSS. At the same time, the qualitative data that resulted from all the methods and instruments were analysed based on the Thematic Analysis of Braun and Clarke [43, 45]. This qualitative analytic method has the goal to identify, analyse and report patterns (named themes) within the data [43, 45]. The first step of the analysis was the familiarization with the data through the transcription and reading of all the verbal data. Then the second phase involved the production of the initial codes through a semantic and inductive analysis. This means that all verbal data relevant to the research questions were coded without any previous framework. Ultimately, the codes were sorted out into themes and sub-themes. The themes capture a pattern that is important for answering the research questions, and the sub-themes give structure to those previous [43, 45].

## Results

### Drug education

The school context was the most stated (65%) as a context that offered drug education followed by television (58%), friends (53.8%) and family (49%). The most addressed topics in school were the consequences when using drugs (98.7%), the dependency (89.3%) and the effects (55.3%). By contrast, the least addressed topics were the precautions to take when using drugs (20%) and information about services that adolescents could access (38.7%).

In relation to the qualitative analyses, in this section, two themes emerged: *The use of drugs as something undesirable; education about drugs as a tool to literacy*. The first theme is divided into two sub-themes. The most important one in this section has a relation to education—*Drug education as a tool for dissuasion*. Of the 143 adolescents that responded to the online questionnaire, 38 emphasized that drug education should focus or reinforce the negative aspects of drug use (“The impact that the use of drugs has in the personal and social life of the users (…) the health effects, the disadvantages” Q134). Five of these participants suggested that the contact with people who use(d) drugs for dissuasion (“People that used drugs (…) to really inform students about the compulsive use of a drug” Q77; “Ex-drug addicts, addicts (…) for the danger message to be received with greater attention” Q121).

On the other hand, in the second theme, the participants had a different position in relation to the role of education on this issue. One of the sub-themes, *transmission of information*, 49 adolescents stated that drug education should include some information that goes beyond the consequences (e.g. what are drugs, the legal framework in Portugal, what kind of support exists) (“what is drug, what is its origin (…)” Q64; “The effects, what to do if the experience goes wrong” Q73). Also, 18 of these participants underlined the need for a *promotion of a safer use* as stated by the following participant:

“In addition to introducing students to the universe of psychoactive substances, addressing risks, the influence of social contexts and that the line between responsible consumption and improper consumption is tenuous and can depend on each person, as well as for the same person under different circumstances. Above all, it should be focused on preparing teenagers who want to experiment, by providing them with the best and most impartial information possible and educating them for responsible drug use.” (Q61)

Another sub-theme is the *relevance of someone with experience in the topic.* Some of the participants (*n* = 37) value the role that someone with direct experience with drugs has in facilitating the sessions about drug education (“real cases revealed by first hand are more appealing” Q45). The close relationship with the audience or being young are two aspects pointed out by one of the participants:

“(…) Imagine, if I go to them [adolescents] and say: “I was born in Matosinhos, in a good house with a pool and without police around”. They will think: “that rich boy, so dumb”. However, if I say: “I was born in [poor neighbourhood of Porto], I sold drugs in another [poor neighbourhood of Porto]”. They will look at me and be more receptive.” (P1)

Inside this sub-theme, professionals also emerge as people with experience. However, their role in drug education is more ambivalent. While 28 of the participants point out the importance of professionals (e.g. doctors, psychologists, police) as promoters of drug education, four of the participants have a different vision about their capability to reach the students. The police intervention is seen as intimidating (“it is facilitated by the police most of the time and this intimidates people to ask questions” Q143; “(…) you are not going to ask how to use drugs to the police” P2). Also the teachers' intervention is seen as contradictory to the students' experience (“The teachers´ perspective tends to not inform the students' ' Q70; “It does not make sense for a teacher who has never used drugs to talk about something that they don't know”).

The other two sub-themes are related to *the role of school* and *the role of family.* In a spontaneous way, 15 adolescents pointed out school as a context for the promotion of drug education and 10 of them said “it should be in the school curriculum” (Q109). Two participants underlined the importance of the family stating that there should exist some form of “education for the parents so that they can raise awareness for the adolescents” (Q20).

### Support for adolescents who use drugs

Of the 143 young people who answered the online questionnaire, 46.2% used some psychoactive substance before the age of 18. However, only one claimed to have attended a service due to their drug use and only 12 of the 133 participants that had used some psychoactive substance knew of any service for drug use in adolescence.

The qualitative theme that emerged was *the perceptions of the adolescents about the need for support when using drugs*, and it has five sub-themes. The first one is *no need for support*. Almost half of the questionnaire participants who had used SPA (*n* = 25) and one participant of the interview, reported that they did not need any kind of support. Some of the reasons they pointed out were: an experimental act (*n* = 5), the absence of difficulties or needs (*n* = 4) and sporadic (*n* = 5) or controlled (*n* = 2) drug use (“Using drugs does not mean that I have had some kind of need or difficulty”. Q39).

In the following two sub-themes there is a focus on the type of need identified: *need for information* and *need for confidentiality in medical intervention*. Regarding the first, about 12 young people in the questionnaire and one of the interviewed participants reported that they needed more information. Four of these participants referred to school as a vehicle for its transmission (“In school, they should have talked openly about drug use, without being so prejudiced against those who experience it” (Q24)). Still, two participants pointed out that information should be transmitted by someone with experience:

“I wish I had that example that I gave you. We were in the room and having a drug user or someone who could explain it well. Because (...) having a teacher saying that you are going to get cancer and some other things; you are not interested.” P1.

In relation to the second mentioned sub-theme, this emerged from the two interviews. Both participants mentioned the fear to look for help in emergency situations (“Most of the adolescents stay in hospital too long due to lack of communication with doctors” P1; “They [adolescents] don't go [looking for help] due to the fear that parents will know” P2).

The last sub-theme is *support from the informal network*. The support of friends (*n* = 7) and family (*n* = 6) was considered very important or because it existed or because of the need for it to be present when they started using drugs. While in friends, support is related to the knowledge they could offer, in the case of the family it is related to the need for openness to communication:

“I think that in many families, drugs are a taboo. This, as in my case, creates a barrier of communication with those who are closest to us and who can often help us more easily. The lack of people of my age who, at the time, were aware and called me to reality, was also a factor that I would have liked to change since they are the people that I would more easily hear and understand. ” Q113

### Data idiosyncrasies

One theme that emerged only in interviews is *the characterization of police intervention*. The first interviewed participant spontaneously referred to police intervention as negative and violent:

“There is a very ugly intervention by the police. Imagine we have adolescents using drugs as cannabis. The police go there and start to apprehend the drug and beat on us. What the police should do is to go there and say: “come here, do you know how bad this is?”. They should try to set an example. Nobody is going anywhere with violence and threats. (...) they don't even earn 5% of people's confidence. If there was more talk and less aggression, they would win more.” E1

The second participant characterized it as something variable and dependent on the agent, but she also referred to some violent episodes.

The last theme, *the vulnerability spectrum*, emerged from the analysis of the focus groups and one of the interviews. The association of the use of drugs with other problems (e.g. poverty, drug trafficking) leads to differences in the typology of drug use among adolescents (“I started selling cannabis at the age of 14. (…) Then I moved on to cocaine and heroin trafficking (…) I was 16 years old…”P1):

“When we place in the equation poverty, marginality, social exclusion and other deviant behaviours, we have a completely different reality compared when these aspects aren´t in the equation.” FG27

### Harm reduction services

In total, 17 of the 143 adolescents claimed to know harm reduction services, but only one claimed to have attended one before adulthood. More than half of the participants considered harm reduction services as a very useful interventional response (52.5%). In relation to the professional’s perspective, only three teams out of 23 considered that the presence of minors is greater than 0% (between 1 and 25%).

In turn, with regard to the analysis of qualitative data*, the use of drugs as something undesirable* as a theme has already been mentioned previously, but the sub-theme to highlight in this section is the perception of *harm reduction as a useless answer to the problem* under study. One of the reasons for its uselessness is pointed out by one of the technicians in the focus group. He emphasizes harm reduction as an assistive response:

“(…) I have some difficulty in realizing today, at this moment, that there is an urge to find solutions for adolescents in terms of harm reduction. (…) We should look for other types of responses and only then can we think about those that are more or less normalized for the adult population” FG21

Another reason presented by 12 of the adolescents who answered the questionnaire is that the focus of the intervention should be to eradicate drug use, especially in those under 18, making this service not a recommended one (“(…) the existence of these services seems to assume that drug use is something that should be considered normal and perpetuated in society”Q117).

On the other hand, *the usefulness of harm reduction services* for this specific issue also emerged from the data. Regarding the sub-theme *promotion of the transmission of information for safer drug use*, 25 adolescents indicated this potential (“It could be useful for young people who don't know how to use drugs. (…) Especially because it is natural for young people who start experimenting to have doubts.” Q61; “This service would better educate adolescents below 18 on this topic as it would really help rather than focus so much on creating fear of the consequences, which happens too much, in my view.” Q70; “[talking about one harm reduction service] “There was a stand to test drugs and the ladies had lollipops (…) they stayed there talking to people, they had little pamphlets and things like that. It was the friendliest intervention I ever saw because the professionals were younger, and the sweets are a good way to start an interaction” P2).

Another theme that emerged was the *absence of minors in the harm reduction teams*. *Stigma as a barrier* is one of the sub themes (“(…) there is also a… stereotype, an idea about the vans. Because the vans are for hangovers” FG22; "I looked at it and thought "if I go there … if someone sees me … I'm going to go through that.". I never wanted to go because that to me was already… if someone saw me, I was going to be considered a drug addict.” E1).

The last sub-theme, *lack of knowledge about how to act*, arises from one of the harm reduction professionals who mentions “(…) we are in a very big ethical dilemma, right? What do we do with these people? They are less than 18 (…) do we give support or don't?” (FG11). This opinion is reinforced by the other professionals in the same focus group who highlight the need for official indications in order to answer this dilemma (“At least some indication from SICAD about what to do in these situations. Not supporting is not an option” FG11).

### Harm reduction and its applicability

The last theme is *harm reduction as a philosophy and not just a service*. This theme is subdivided into two sub-themes that aim to structure the suggestions listed. The first sub-theme is *the extension of harm reduction intervention.* There is a need to change the standard model of intervention mentioned by one of the professionals in the field:

“(…) The model we have of intervention is a model made in the image of what was thought to be our typical client, isn't it? White male, middle class, drug user (…) all the research that I am seeing points to the need to deconstruct this model (…) deconstruct this to understand how, within a universal model, we can find specificities for men, women, older drug users, younger drug users” FG27

Also within this sub-theme, one participant referred the application of this philosophy to alcohol use and one of the interviewees stressed the need to adapt existing responses to the constraints of being less than 18 years old: “you have to take into account all the things that entails not being 18 years old such as living with your parents, having school schedules, not going out so easily, not wanting your parents to know you went into an alcoholic coma last Friday.” (E2).

The other sub-theme is *the diversification of the application of harm reduction to different contexts*. In total, 25 of the young people who answered the questionnaire and one of the interviewees referred to school as a context for applying this philosophy and four referred the application of this through an intervention in the informal network.

## Discussion

In order to facilitate the reading and comprehension of the previous section, the discussion is organised into three topics associated with the research question as demonstrated in Table 1.

**Table 1:** Association between the research questions, the themes and the topics of the discussion

Topics of the discussion	Goal of each research question	Connection with the theme and subthemes of the thematic analyse	
The needs of the young people who use drugs and their view about the existing services	2b—Youngster’s perspective about how their need should have answered	The perceptions of the adolescents about the need for support when using drugs	No need for support
			Need for information
			Need for confidentiality in medical intervention
			Support from the informal network
		The vulnerability spectrum	–
The importance of education about drugs	2a—Youngster’s perspective about education about drugs in formal and informal contexts	The use of drugs as something undesirable	Drug education as a tool for dissuasion
		Education about drugs as a tool to literacy	Transmission of information
			Relevance of someone with experience in the topic
			The role of school
			The role of family
	2c—Youngster’s perspective a out their vision about services and institutions	The characterization of police intervention	–
Harm reduction—its potential	1. The perspective of Harm Reduction Professionals about: the dimension, the needs and the obstacles that adolescents´ drug users face when using harm reduction services	Harm reduction as a useless answer to the problem	–
		Absence of minors in the harm reduction teams	Stigma as a barrier
			Lack of knowledge about how to act
	3—The relevance of Harm Reduction philosophy	The usefulness of harm reduction services	Promotion of the transmission of information for safer drug use
		Harm reduction as a philosophy and not just a service	The extension of harm reduction intervention
			The diversification of the application of harm reduction to different contexts

### The needs of the young people who use drugs and their view about the existing services

In congruence with the literature, the lack of access to institutions may be due to the lack of knowledge about them [16, 24] as demonstrated in the data where few participants refer to any known service.

On the other hand, many of the young people surveyed claimed to have not felt any need for support. The characterization of drug use as "controlled" or "sporadic" does not allow them to identify themselves as drug users transforming the services that addressed this problem as irrelevant to them [16, 36].

Furthermore, the view of services with the function of providing support seems to be interpreted by some of the adolescents with a unique focus—solving problems directly related to drug use. The potential for other purposes that services have is not observed in the young people's perspectives. However, most adolescents identified the need for information, revealing a reduced focus of the current interventions in the empowerment of decision-making, which is based on the acquisition of information [25, 26].

Another need that arose in the interviews with young people who have used drugs under the age of 18 is confidentiality in medical interventions. The fear of nonconfidentiality, an aspect also highlighted in the literature [22, 24], may be an obstacle for seeking services, particularly in situations of discomfort.

Finally, the informal network is seen as an important source of support when it is present and necessary when it is absent. Both friends and family appear as a source to transmit knowledge and enhance dialogue. Thus, the exclusive focus on the individual and on individual intervention is insufficient [39, 40]. There seems to be a pertinence of intervening in informal networks to empower them with important tools to meet the needs of young people who use drugs.

### The importance of education about drugs

The school is the most referenced context for drug education by young people, followed by family and friends, gaining the informal network again prominent in the data.

One of the interviewees referred to the students' low receptivity in drug education contexts, pointing out the interlocutor as one of the reasons. In this study it emerged the contrasting views of young people in relation to the professionals who run school sessions. The perspectives that view the professionals as facilitators suggest that for these young people, authority presents itself as a relevant source for obtaining safe information. This may be related to the nonuse of drugs before the age of 18. On the contrary, the use of psychoactive substances before the age of 18 may be one of the reasons why some adolescents do not recognize the usefulness of the information transmitted by these same professionals. In this perspective, the role of a peer, someone with personal experience on the topic or close to the age of the target audience, emerges as a relevant figure in drug education.

Finally, these school sessions are marked by a strong focus on consequences, which demonstrates that drug education continues with an approach that emphasises prevention and abstinence [13, 15, 31, 37, 38] despite its ineffectiveness being continuously proven [15, 31, 33]. If this is the actual focus on drug education, the need for information referred to in the previous point and identified by most of the participants may suggest that they seek other types of information. This draws attention to the importance of youth involvement in the development of the topics addressed in the school sessions.

One of the advantages of this study is the fact that it took into account the perspectives of young people, whether or not they used drugs before the age of 18. This has allowed personal reflections on the phenomenon, namely the focus that drug education should have. While a group of adolescents consider that it should be used as a deterrent to the use of drugs, seen as something undesirable, another group advocates drug education as a tool for literacy, highlighting the promotion of safer drug use that can constitute the information that young people who use drugs need. As a result, discourses that focus on abstinence, despite being accepted by a segment of the youth population, isolate those who use(d) drugs and those who have a different personal perspective [32]. This conclusion highlights the need to tailor the interventions to the diverse experiences with drugs instead of interventions “one size fits all”.

### Harm reduction: its potential

Few teams report the attendance of adolescents under the age of 18 as users of harm reduction services, something common according to the literature [16, 35, 37]. However, despite the long-standing existence of the harm reduction services, their intervention with this population is neither exact nor easy to define, as it remains in legal limbo [21, 34].

The stigma of going to a mobile harm reduction service and the lack of knowledge about this service [16] seems to be the two main reasons for the almost nonexistent presence of young people. Few adolescents reported knowing about this service, demonstrating that, even in recreational contexts, it may not be available or may not be reaching this group.

The capacity that harm reduction services have to be more than a support service, assuming itself as a philosophy, arises from the responses of the various participants and professionals. They highlight the need for harm reduction intervention to be more comprehensive and exceed what the typical model of intervention is and the typical drug user (e.g. male, middle aged with a problematic use of heroin and cocaine) [16, 34, 36, 37].

In the perspective of young people, whether they know harm reduction services or not, this services philosophy can be applied in other contexts being school the most mentioned one. The need to increase the spectrum of knowledge of young people regarding drugs can be answered by the scaling-up of harm reduction interventions. Despite not being mentioned as widely as the previous one, the informal network is also identified as a context to be taken into account in the dissemination of harm reduction principles allowing this network to be effectively and actively present for young people who use drugs when they are underage.

## Conclusion

Throughout this exploratory study, we seek to achieve a closer understanding of the interventional needs, the existing responses, as well as those that are necessary to create for adolescents who use drugs.

It should be noted that this study also has some limitations. The first to emphasize is the fact that it is a retrospective study, not allowing people under the age of 18 to be the participants. On the other hand, although the questionnaire involves open questions, the qualitative dimension did not have the desired and necessary depth to understand the individual narratives. Although extending the questionnaire to any young person between 18 and 25 years old has brought advantages and new views of the phenomenon, the needs of young people who use drugs under 18 are different from those who do not use them. In fact, we consider that a more detailed exploration of this same phenomenon would be pertinent, in order to obtain the perception of young people under the age of 18 who use drugs in relation to the aspects developed in this research.

Therefore, the issue under analysis does not end with this document, but it is possible to point out relevant conclusions to be deepened and developed in future investigations and projects. In general, this investigation, by covering all adolescents, independently if they have used or not used drugs under the age of 18, allowed us to verify that there is a spectrum of the type of users and their drug use [46, 47] which makes each young person’s vulnerability distinct.

There are young people who will not use drugs before the age of 18, others where this use will not be accompanied by problems or that will have the necessary support. Others will present some problems and may have associated with the drug use, other social issues (e.g. poverty, social exclusion, drug trafficking) that add to the vulnerability of being under 18 and many other vulnerabilities derived from the different social circumstances in which they found themselves [12, 31].

Many dimensions of the phenomenon remained to be addressed, such as the differences resulting from diverse social circumstances. However, it was a study that took the first steps in identifying the vulnerabilities of being under 18 and using drugs [34, 37, 38]. Empowering means giving knowledge, the greatest need listed by the majority of young people surveyed, and the school may be the starting point for an intervention based on the harm reduction philosophy in which young people emerge as receivers and deliverers by being elements of an informal network of other adolescents at the moment and in the future as adults.

## Data Availability

The datasets used and/or analysed during the current study are available from the corresponding author on reasonable request.

## Footnotes

1 The research questions were constructed based on the acronym PICo. In qualitative research PICo stands for Population, Interest (that relates to a defined event, activity, experience or process) and Context.

2 Harm reduction teams are multidisciplinary teams with the goal to promote harm reduction strategies by intervening in the public space where drug use is experienced as a social problem.
